# Extracellular Vesicles Derived From *Citrus sinensis* Modulate Inflammatory Genes and Tight Junctions in a Human Model of Intestinal Epithelium

**DOI:** 10.3389/fnut.2021.778998

**Published:** 2021-11-24

**Authors:** Stefania Paola Bruno, Alessandro Paolini, Valentina D'Oria, Angelo Sarra, Simona Sennato, Federico Bordi, Andrea Masotti

**Affiliations:** ^1^Research Laboratories, Children's Hospital Bambino Gesù-IRCCS, Rome, Italy; ^2^Microscopy Center, University of L'Aquila, L'Aquila, Italy; ^3^CNR-ISC UOS Sapienza and Department of Physics, Sapienza University of Rome, Rome, Italy

**Keywords:** fruit-derived extracellular vesicles, *Citrus sinensis*, intestinal epithelium, intestinal permeability, inflammatory stimulus

## Abstract

It is widely acknowledged that mammalian exosomes (or extracellular vesicles), have a key role in intercellular communication, owing to the presence of various bioactive molecules such as lipids, proteins, and microRNAs within their inner compartment. Most recently, the discovery of extracellular vesicles isolated from edible plants (such as vegetables and fruits) and their similarity in terms of size and content with exosomes has opened new perspectives on possible intercellular communication and regulation of important biological processes in which these vesicles are involved. It is also well-known that a balanced diet rich of fruits and vegetables (i.e., the Mediterranean diet) can contribute to maintain a “healthy gut” by preserving the intestinal epithelial barrier integrity and avoid that inflammatory stimuli that can alter homeostasis. In our study, we optimized a method to isolate extracellular vesicles from the orange juice *(Citrus sinensis)* (CS-EVs), and we characterized their morphology and behavior when in contact with the intestinal epithelium. We showed that CS-EVs are stable in a simulated gastrointestinal environment and are absorbed by intestinal cells without toxic effects, as expected. Furthermore, we demonstrated that CS-EVs can alter the gene expression of several genes involved in inflammation (i.e., *ICAM1* and *HMOX-1*) and tight junctions (i.e., *OCLN, CLDN1*, and *MLCK*), contributing to limit inflammatory stimuli and restore a functional barrier by increasing the tight junction OCLN protein. Therefore, our study emphasizes the relevant role of fruit-derived extracellular vesicles in modulating important biological processes and maintaining a healthy intestinal epithelium, ultimately promoting human health and well-being.

## Introduction

In the last several years, many studies about mammalian extracellular vesicles (EVs) have confirmed the role of these small vesicles as crucial determinants of intra- and intercellular communication (1–3). EVs have the ability to mediate cell-to-cell communication by transferring to recipient cells a variety of molecules able to regulate gene and protein expression (4, 5). EVs represent a family of membranous vesicles of different sizes (ranging from 30 nm up to 5 μm) released by different mammalian cells (6), and, among them, exosomes have recently received great attention for their potential in diagnostic and therapeutic applications (7, 8).

Exosomes are endocytic-origin nanovesicles with a diameter of 30–100 nm, which were observed in biological fluids of different organisms. The internal exosomal compartment generally contains various bioactive molecules, such as DNA, proteins, lipids, coding and non-coding RNAs, and microRNAs (miRNAs), etc. (9, 10).

More recently, a growing interest (and also controversial) has focused on the discovery of “exosome-like” extracellular vesicles in edible plants (i.e., vegetables, fruits, etc.) (9, 11–14). In fact, many proteins of *Citrus limon* EVs overlapped with mammalian exosomes proteins or belong to functional groups that characterize exosomes regardless of their cellular origin (9). The increasing number of studies about the isolation protocols and the characterization of these nanosized particles indicates that the interest in this field is growing as well, owing to the potential use of these EVs in many fields (i.e., delivery of therapeutic molecules). In particular, EVs have been isolated from tomatoes (15), grapefruit and grape juice, carrots, and ginger (11, 12, 16), lemon (9), and sunflower seeds (13) through differential centrifugation and ultra-centrifugation steps. Generally, these protocols have been adopted from those employed to recover mammalian EVs and exosomes.

Several studies suggested that plant-derived nanosized particles are also able to regulate cell homeostasis (17–19). Moreover, Zhang et al. have demonstrated that nanoparticles derived from grape, ginger, grapefruit, and carrots have anti-inflammatory properties in severe intestinal diseases [i.e., inflammatory bowel diseases, IBDs (11)]. Similarly, the anti-inflammatory role of plant-derived EVs was also found for broccoli-derived vesicles, which were able to target intestinal dendritic cells (DCs) and activate adenosine monophosphate-activated protein kinase (AMPK), which prevented DCs activation and induced tolerant DCs in three different models of colitis in mice (20). Inflammatory bowel diseases are characterized by an epithelial barrier dysfunction and increased intestinal permeability, thus representing a perfect example of dysregulated cell homeostasis (17). A diet rich of fruit and vegetables has been demonstrated to have a great influence on the regulation of mammalian host cell homeostasis (18, 19), suggesting a pivotal role of vegetable components (and maybe also of their EVs content) in modulating the intestinal permeability and its integrity (38).

During the inflammatory process, pro-inflammatory cytokines, such as TNF-α, IFN-γ, IL-1β, and IL-8, have been reported to cause a dysfunctional opening of intestinal junctions (i.e., tight junctions, TJ) and an increase of the intestinal permeability (21–24). In this regard, several studies have focused on myosin L chain kinase (MLCK) that plays a key role in the regulation of intestinal TJ permeability (22, 23). The activation of MLCK catalyzes the phosphorylation of myosin L chain (MLC), which induces a contraction of peri-junctional actin-myosin filaments and opening of the TJ barrier (25). Therefore, the inhibition of MLCK activation prevents the increase in intestinal TJ permeability. It is still unknown whether extracellular vesicles from edible plants could play an active role *in vivo* in the improvement of the intestinal barrier function after an inflammatory stimulus, but a regulation of *MLCK* gene by EVs could be an innovative opportunity for the treatment of this kind of dysfunction.

To study in more detail the regulatory role of fruit-derived EVs, in our work, we characterized extracellular vesicles isolated from sweet oranges (species *Citrus sinensis*; variety, *Tarocco*) (CS-EVs), a fruit commonly included (and consumed) in the Mediterranean diet. We demonstrated that CS-EVs are abundant in this fruit and that they are stable under environmental conditions that mimic the gastrointestinal tract. Moreover, we demonstrated that CS-EVs are able to penetrate into intestinal epithelial cells and modulate positively the expression of anti-inflammatory genes and tight junctions. Therefore, although additional specific research is needed, our work supports the concept that CS-EVs may have beneficial effects also *in vivo*, once consumed regularly by following a balanced diet (i.e., Mediterranean diet).

## Materials and Methods

### Isolation of Extracellular Vesicles From Sweet Oranges (*Citrus sinensis*)

Extracellular vesicles were isolated from sweet oranges of the species *Citrus sinensis* (CS-EVs). Fresh fruits were purchased from a local producer (certified origin), carefully washed and manually squeezed. The orange juice (350 ml obtained by squeezing approximately four oranges) was mixed with an equal volume of 5% glucose solution and filtered through a paper filter and then through a 0.45-μm membrane filter. To remove residual debris, the juice was finally centrifuged at 800 g for 20 min. To concentrate the juice, the supernatant was ultrafiltered by Vivaflow 200 cell (Sartorius) and ultracentrifuged at 100,000 g for 3 h. The pellet obtained after the ultracentrifugation was washed one time with PBS and ultracentrifuged for additional 3 h. The washed pellet was resuspended in PBS, and protein content was determined by Pierce™ BCA Protein Assay Kit (Thermo) according to the manufacturer's protocol. CS-EVs were finally stored at −20°C until use.

### Extracellular Vesicles (CS-EVs) Identification by Dynamic Light Scattering (DLS)

Dynamic light scattering was employed to identify extracellular vesicles contained in the sample. The size distribution of CS-EVs was determined after diluting the vesicle samples with PBS to avoid possible aggregation between the particles by employing a Nano Zetasizer apparatus equipped with a 5 mW HeNe laser (Malvern Instruments LTD, UK). This system uses backscatter detection, i.e., the scattered light is collected at 173°. To obtain the size distribution, the measured autocorrelation functions were analyzed by means of the CONTIN algorithm (26). Decay times are used to determine the distribution of the diffusion coefficients D_0_ of the particles, which, in turn, can be converted in a distribution of apparent hydrodynamic diameter, D_h_, using the Stokes-Einstein relationship D_h_ = k_B_T/3πηD_0_, where k_B_ is the Boltzmann constant, T the absolute temperature, and η the solvent viscosity. The values of the radii shown in this work correspond to the average values on several measurements and are obtained from intensity-weighted distributions (26).

### Extracellular Vesicles Characterization by Transmission Electron Microscopy (TEM)

Transmission electron microscopy was employed to characterize the morphology of CS-EVs. All of the TEM measurements have been performed by depositing 20 μl of suspension of vesicles on a 300-mesh copper grid for electron microscopy covered by a thin amorphous carbon film. Samples have been deposited at room temperature. Negative staining was realized by addition of 10 μl of 2% aqueous phosphotungstic acid (PTA) solution (the pH was adjusted to 7.3 using 1-M NaOH). Measurements were carried out by using a FEI TECNAI 12 G2 Twin (FEI Company, Hillsboro, OR, USA), operating at 120 kV and equipped with an electron energy loss filter (Biofilter, Gatan Inc, Pleasanton, CA, USA) and a slow-scan charge-coupled device camera (794 IF, Gatan Inc, Pleasanton, CA, USA).

### Assessment of Extracellular Vesicles Stability in Simulated Gastric Environment

The evaluation of CS-EVs stability in the gastrointestinal tract was carried out by incubating vesicles (100 μl) with three different solutions that mimic the gastric and the intestinal environment. For the simulation of the gastric solution (GS), pepsin (3 g/L) (from porcine stomach mucosa, Sigma-Aldrich) and lipase (0.9 mg/L) (lipase G from *Peniciliumcamemberti*, Sigma-Aldrich) in PBSat pH = 2–2.5 were mixed with CS-EVs. After the measurements in GS, small aliquots of an alkaline solution (150 ml of 1MNaOH and 14 g of NaH_2_PO_4_·2H_2_O in 1 L of distilled water) were added to increase the pH value. To mimic the environment of the first intestinal tract (EP1, enteric phase 1), bile (100 mg/ml) (bovine bile, Sigma-Aldrich) and pancreatin (10 mg/ml) (from porcine pancreas, Sigma-Aldrich) were dissolved in PBS, and pH was adjusted to 5.4–5.7. The solution for the second intestinal tract (EP2, enteric phase 2) was prepared starting from EP1 and adjusting the pH to 6.8–7.2 with the same alkaline buffer employed to prepare EP1. After 2-h incubation, DLS was employed to monitor vesicles dimension and evaluate aggregation and degradation.

### Cell Culture Model

The human colonic epithelial cell line CaCo-2 was cultured in DMEM (Dulbecco's modified MEM) High Glucose (Life Technologies) supplemented with 10% fetal bovine serum (FBS) (HyCloneFoetal calf serum), 1% non-essential amino acids, 1% penicillin-streptomycin, and 1% pyruvate sodium. Cell culture was incubated at 37°C in a 5% CO_2_ atmosphere. Cells were routinely subcultured one time a week with Trypsin-EDTA solution and seeded at density of 4.5 × 10^3^ cells/cm^2^ to reach 50% confluence on Day 4 (~5.5 × 10^4^ cells/cm^2^). For differentiated CaCo-2 cells, the epithelial cells are seeded on filter cell culture chamber inserts (Transwell with polycarbonate filters and 0.4-um filter dimension, Greiner Bio-One) at a density of 3 × 10^5^ cells/cm^2^. This seeding density allowed to reach confluence within 48 h. When confluence was reached, we employed a cell culture medium supplemented with 10% FBS for BL (basal) and without FBS for AP (apical) compartments. The medium was changed three times a week. A full differentiation is generally observed after 21 days after cell seeding. To assess the differentiation process, we monitored the trans-epithelial electrical resistance (TEER) with a Millicell ERS-2 Volt-Ohm meter (Millipore) equipped with a special electrode having the measuring tips covered by a silver/silver chloride (Ag/AgCl) pellet on each electrode tip. In our conditions, values higher than 1,200 Ohm·cm^2^ corresponded to complete differentiation.

### Cytotoxicity Assay (MTT Assay)

Cell viability was measured by using the MTT [3-(4,5-dimethylthiazol-2-yl)-2,5-diphenyl tetrazolium bromide] assay on CaCo-2 cells. Briefly, cells were seeded at a density of 0.1 × 10^5^ in a 96-well-plate and incubated with an increasing dose (from 5 to 20 μg/ml) of CS-EVs for 24, 48, and 72 h at 37°C. After incubation, the medium was removed and the cells were thoroughly rinsed with PBS. The cells were incubated with 50 μl of MTT (5 mg/ml) for 2 h until a purple precipitate was viewed. Then, the solution was replaced with 50-μl dimethyl sulfoxide (DMSO) in each well prior to spectrophotometric measurements at 570 nm. Means and standard deviations generated from three independent experiments were reported as percentage of viable cells vs. control (untreated) cells.

### *In vitro* Internalization of CS-EVs by CaCo-2 Cells

The CaCo-2 cells system described above allowed to evaluate the ability of CS-EVs to penetrate through the intestinal layer or of the epithelium to internalize them, thus mimicking the *in vivo* behavior. To visualize CS-EVs, after isolation performed as described above, vesicles were labeled with Alexa Fluor™ 647 NHS Ester (ThermoFisher). Briefly, the NHS-modified dye was solubilized in dimethyl sulfoxide (DMSO) at a concentration of 10 mg/ml, and 3 μl was added to 100 μl of CS-EVs (200 μg) in PBS/0.1-M NaHCO_3_ (pH 8.3) (100 μl). Vesicles were incubated with the dye at room temperature for 1 h. Labeled vesicles were purified by a filter column (Centrifugal Filter Units-Amicon Ultra, 10000 NMWL), washed three times with sterile water, and, finally, suspended in sterile PBS. Undifferentiated and differentiated CaCo-2 cells were treated with 5 μg/ml of Alexa-labeled CS-EVs for 6 and 24 h at 37°C. After incubation, cell membranes were stained with WGA-488 FITC (green) (1:200 dilution) for 20 min in the dark, and fixed with 4% paraformaldehyde (PFA) for 20 min. After washing with PBS, nuclei were stained with Hoechst 33342 (Molecular probes, Life Technologies) (1:2,500 dilution) for 3 min in the dark, and then the cells were rinsed thoroughly with PBS. An Olympus Fluoview FV1000 confocal microscope (Olympus, Italy) equipped with FV10-ASW version 4.1a software, Multi Ar (458–488 and 512 nm), 2X He/Ne (543 and 633 nm), and a 405-nm diode laser, using a 40 × (N.A.90) objective and a 60 × (N.A 1.42) oil objective, was employed to visualize slides.

### Immunofluorescence and Tight Junction Evaluation

Differentiated CaCo-2 cells were treated with the inflammatory cocktail (TNF-α, IL-1β, and IFN-γ), with or without 20 μg/ml pretreatment with CS-EVs for 48 h. The inflammatory cocktail was applied to the basolateral compartment at the concentration of 10 ng/ml per each compound. At the end of these treatments, CaCo-2 cells were washed with PBS, fixed with PFA 4% for 15 min, and rinsed three times with PBS. For occludin staining, the cells were fixed with a Blocking Buffer (1 × PBS/5% normal serum/0.3% Triton™ X-100) for 1 h. The cells were then incubated with a rabbit anti-occludin antibody diluted 1:200 in a dilution buffer (1 × PBS/1% BSA/0.3% Triton™ X-100). Primary antibodies were incubated overnight at 4°C. The cells were then washed again with PBS and incubated with the secondary antibody Alexa Fluor 488 goat anti-Rabbit (1:500) for 1 h at room temperature in the dark. The cells were then washed again with PBS and excised from the Transwell insert using a scalpel. The permeable support membrane was then mounted cell side up between a slide and a coverslip with a mounting medium containing Hoechst 33342. CaCo-2 cells were acquired with a Leica TCS-SP8X laser-scanning confocal microscope (Leica Microsystems, Mannheim, Germany) equipped with a tunable white light laser (WLL) source, a 405-nm diode laser, three Internal Spectral Detector Channels (PMT), and two Internal Spectral Detector Channels (HyD) GaAsP, 10 X, and 20 X (NA 0.4 and 0.7) magnifications. Z-reconstructions of serial single optical sections were performed with a sequential scanning mode of 1,024 × 1,024 pixels, scan speed of 400 Hz, and z-step size of 2.5 μm.

### RNA Isolation and Quantitative Real-Time Polymerase Chain Reaction (qPCR)

CaCo-2 cells were seeded into 24-transwell inserts and grown up to differentiation. The trans-epithelial electrical resistance (TEER) was monitored periodically by using a Millicell ERS-2 apparatus (Millipore). After 21 days, the cells were completely differentiated and were pretreated with 20 μg/ml of CS-EVs for 24 h. The cells were stimulated with a cocktail of inflammatory cytokines (TNF-α, IL-1β, and IFN-γ) at two time points (6 and 24 h). TNF-α, IL-1β, and IFN-γ were applied to the basolateral compartment at a concentration of 10 ng/ml each. After treatment, the cells were collected, and total RNA was extracted by the Total RNA Purification Plus Kit (Norgen, Biotechnologies Co., Canada), according to the manufacturer's specifications. Total RNA (1 μg) was reversely transcribed to cDNA using the SuperScript III Reverse Transcription Kit (Invitrogen) in a final volume of 20 μl following the recommendations of the manufacturer. cDNA samples were amplified by quantitative real-time PCR (qPCR) by using the SensiFAST Probe Lo-ROX master mix (Bioline) and specific Taqman primers (IDT Technologies) for human IL-6, ICAM-1, HMOX-1, MAPK-1, CLDN-1-4, OCLN, TJP1, GJB3, REG3G, SRC, CTSB, MYLK, and TLR8. The gene-specific primers selected are listed in Supplementary Table 1. The expression of housekeeping gene GUSB was used to normalize the expression levels of target genes, and the relative expression levels were calculated by using the 2^−ΔΔCt^ method.

### Statistical Analysis

Statistical comparison between various groups was performed by Student's *T*-test or one-way ANOVA with least significant difference (LSD) *post-hoc* tests, using the SPSS software (12.0.2). Comparisons were made between means from several experiments. Differences were considered significant when *p*-values were <0.05. Statistical significance is indicated as^*^ for *p* < 0.05 and ^**^ for *p* < 0.01.

## Results

### Sweet Orange (*Citrus sinensis*) Juice Contains Extracellular Vesicles (CS-EVs)

**Figure 1:**
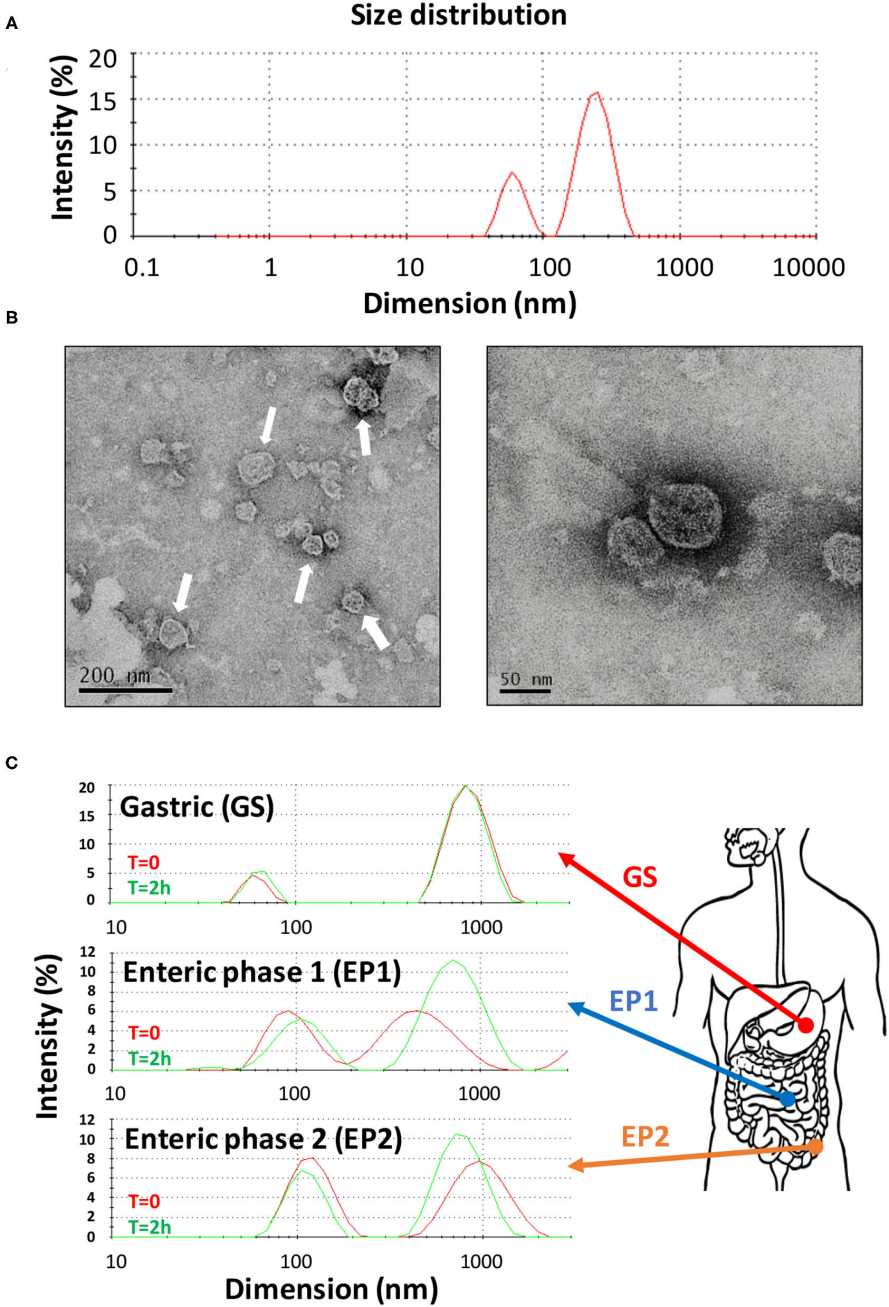
Characterization of CS-EVs. Size distribution of CS-EVs in PBS **(A)** measured by DLS. TEM images of a preparation of CS-EVs **(B)** with a magnification of single vesicles. Simulated gastric solutions were employed to assess the stability of CS-EVs and mimic their behavior in the gastrointestinal tract **(C)**.

*Citrus sinensis* extracellular vesicles were isolated from the orange juice by performing several filtration, ultrafiltration, and ultracentrifugation steps by following a procedure that greatly improved the recovery of vesicles (27). In fact, starting from only 350 ml of initial orange juice, we obtained preparations of CS-EVs with a final protein concentration of 1 mg/ml. The procedure that we optimized had some advantages: the ultrafiltration procedure allowed us to concentrate the initial solution up to 40–50 ml, whereas the ultracentrifugation step allowed us to recover visible pellets further purified by several washing steps. By following this protocol, we obtained a uniform and purified preparation of CS-EVs. The diffused light intensity graph of CS-EVs sample obtained by dynamic light scattering (DLS) showed a bimodal distribution, indicative of two different vesicle populations (Figure 1). The diameter of the smallest population was 62 ± 12 nm, whereas the biggest vesicles measured 247 ± 61 nm (Figure 1A). Since the intensity of the scattered light is proportional to the sixth power of the diameter, even small traces of larger objects give a high contribution to the intensity compared to smaller particles. To have a closer look at the morphology of these vesicles, we performed a TEM analysis that confirmed the presence of vesicular structures. In particular, we confirmed not only that the size of observed vesicles was comparable to that obtained by DLS data (Figure 1B), but that these vesicles tend to aggregate and form larger particles (white arrows in Figure 1B). These data suggested that the size distribution observed by DLS can be the consequence of the aggregation of small particles. Moreover, DLS and TEM analysis suggested that CS-EVs have the right dimension and morphology to be classified as “exosome-like” extracellular vesicles.

### CS-EVs Are Stable in *in vitro* Models That Mimic the Gastrointestinal Tract

Our hypothesis is that CS-EVs may have a beneficial effect when they come in contact with the epithelial cells of the gastrointestinal tract, translocate into them, and, eventually, deliver their cargo (i.e., nucleic acids and proteins). However, to have an effect *in vivo*, CS-EVs should pass intact the gastrointestinal tract and remain unaltered to the extreme environmental conditions (i.e., low pH, presence of proteolytic enzymes, etc.). Therefore, to assess whether CS-EVs are stable in all the gastrointestinal tract, we prepared three different aqueous solutions, with a composition resembling that of the three main gastrointestinal tracts (i.e., stomach, duodenum, and colon). These solutions (i.e., GS, EP1, and EP2, respectively) were then used to incubate CS-EVs for 2 h. Size distribution of vesicles was recorded at the end of the incubation for each solution. Figure 1C shows that CS-EVs incubated in the gastric solution (GS) maintained their initial dimension (from 61 ± 8 to 64 ± 9 nm, *p* > 0.05 and from 873 ± 207 to 852 ± 193 nm, *p* > 0.05). Similarly, not statistically significant results were also obtained for the remaining two enteric phase solutions during the 2-h incubation period. The sequential incubation of CS-EVs in the enteric phase 1 (EP1) solution showed a significant dimensional increase of the smaller population compared with the gastric solution (from 63 ± 9 to 98 ± 29 nm, *p* < 0.05), whereas the difference between EP1 and EP2 (from 98 ± 29 to 108 ± 30 nm) was not statistically significant (Figure 1C). The larger population did not display significant dimensional differences in the three solutions. Overall, we can emphasize that CS-EVs are dimensionally stable when incubated in GS, EP1, and EP2 but slightly increase their dimension when they are incubated in EP1/EP2 compared with GS.

### CS-EVs Are Not Cytotoxic and Are Easily Internalized by Intestinal Cells

**Figure 2:**
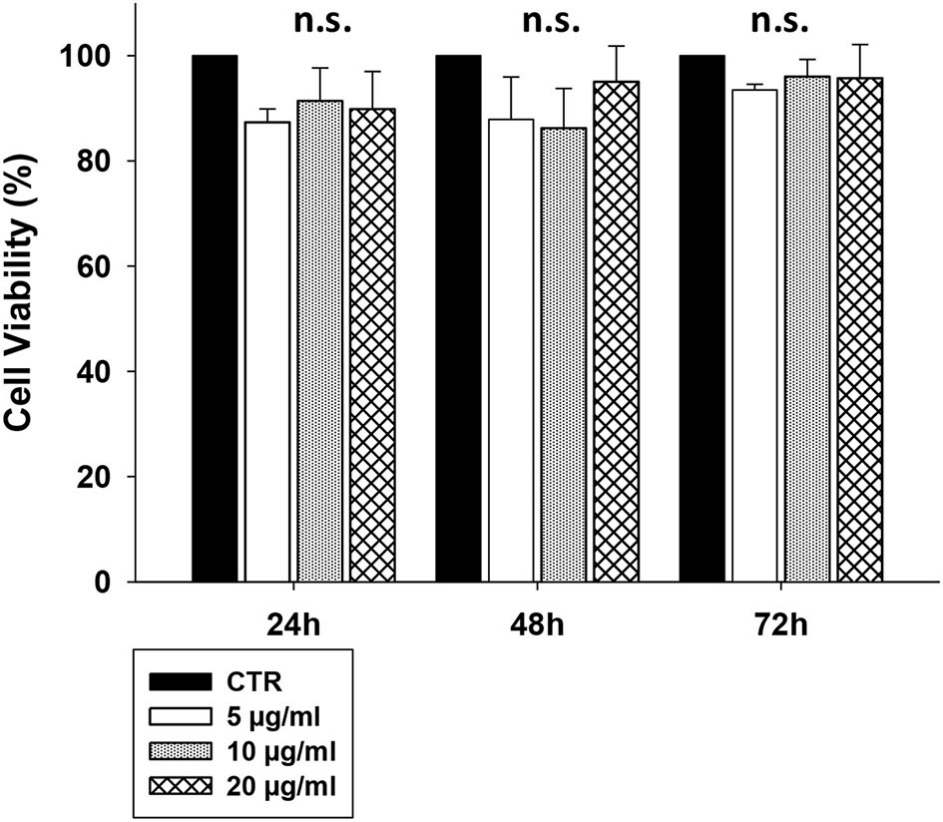
CaCo-2 cells viability after CS-EVs treatment. Cell viability was assessed by MTT assay at 24, 48, and 72 h after incubation with 5, 10, or 20 μg/ml of CS-EVs. Each histogram represents the mean ± SD of three independent experiments.

To assess whether CS-EVs have cytotoxic effects on epithelial cells, confluent CaCo-2 cells were treated with vesicles at increasing concentrations (from 5 to 20 μg/ml). The MTT viability assay showed that the treatment with 5, 10, or 20 μg/ml of CS-EVs did not alter significantly the cell viability compared with untreated cells (Figure 2).

**Figure 3:**
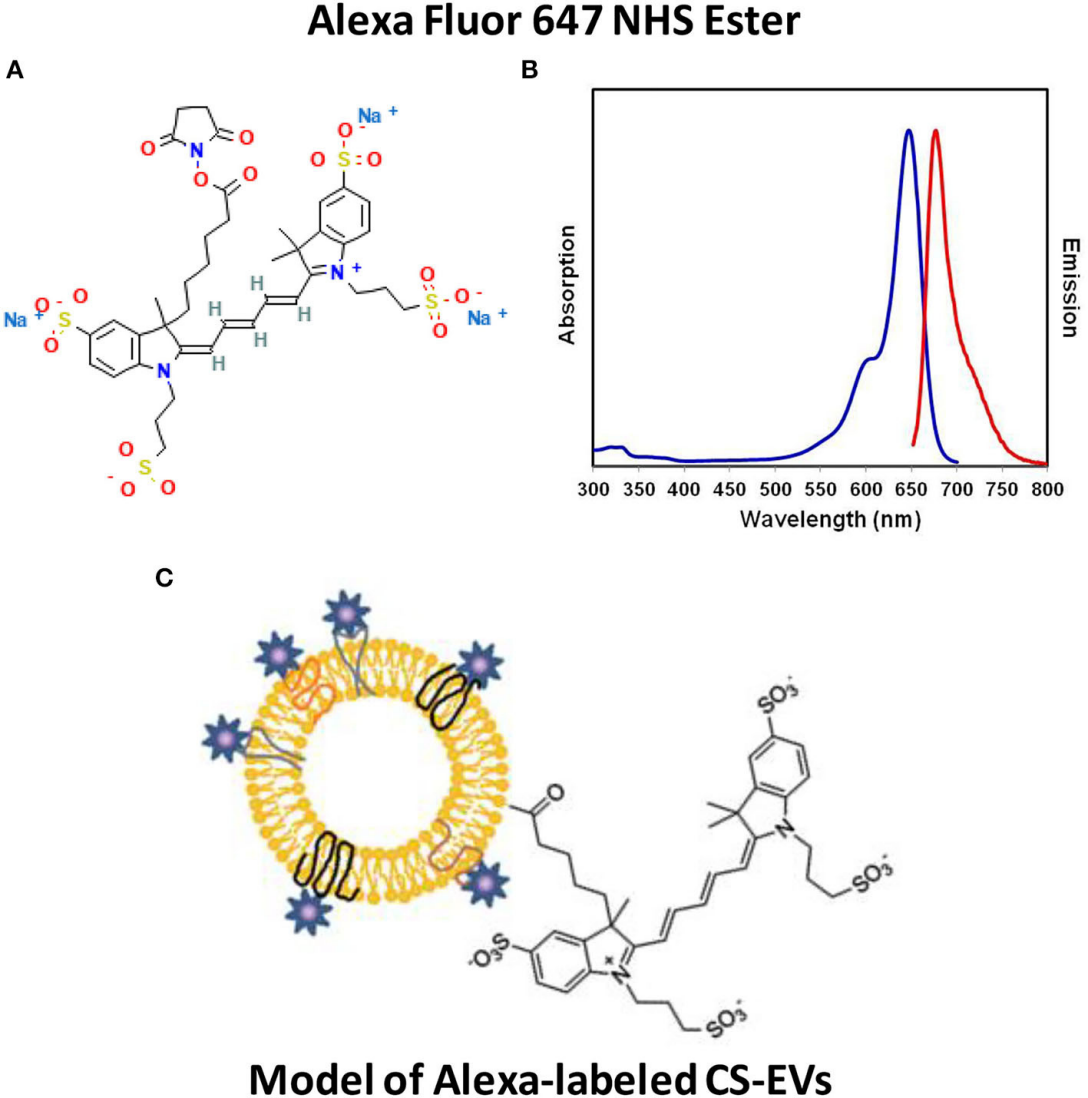
Functionalization of CS-EVs with Alexa™ fluor 647 dye. Alexa fluor 647 NHS ester structure **(A)** with spectral characteristics **(B)**. CS-EVs can be efficiently labeled by following a simple bioconjugation protocol **(C)**.

**Figure 4:**
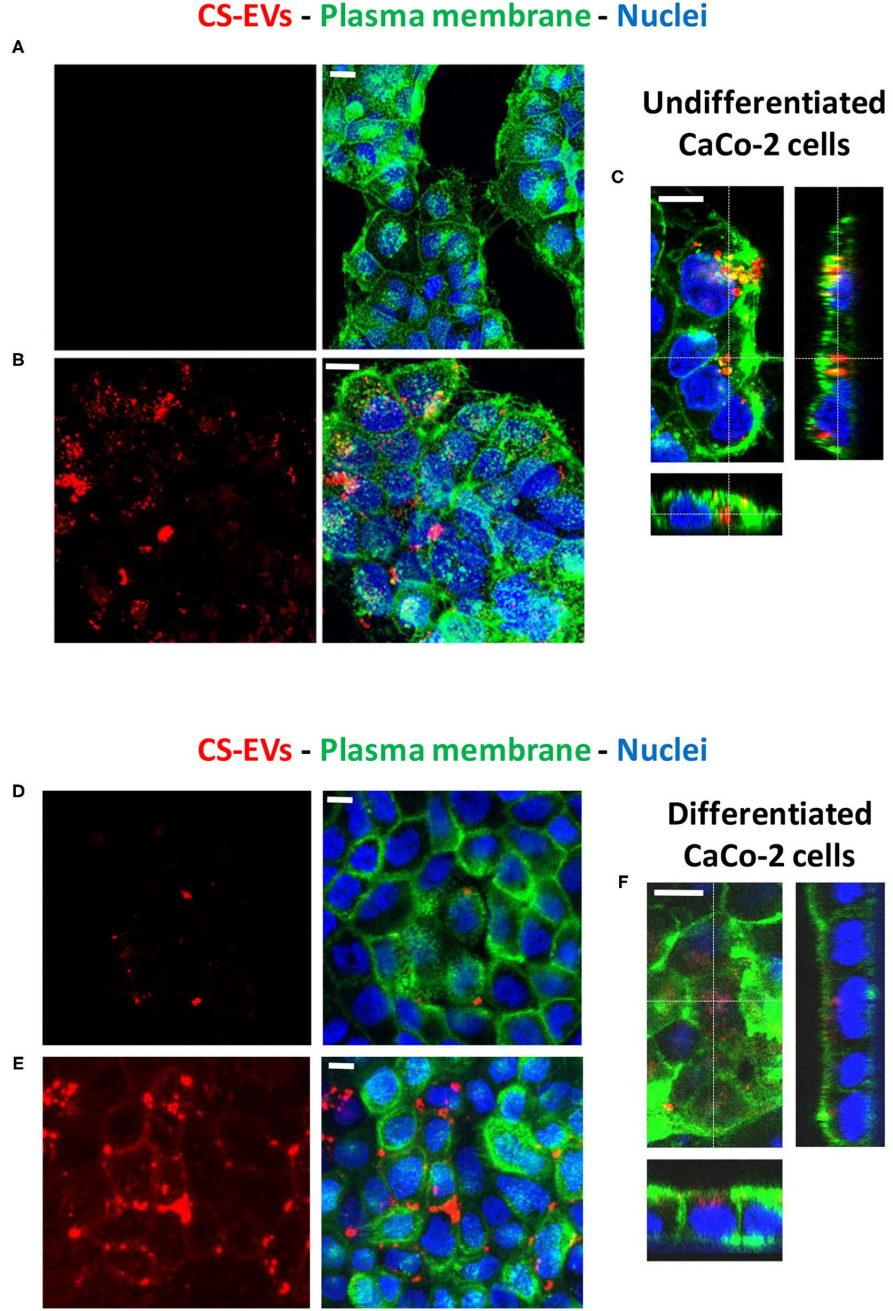
Uptake of CS-EVs by intestinal epithelial cells. **(A)** Undifferentiated CaCo-2 cells. **(B)** Undifferentiated CaCo-2 cells treated with 5 μg/ml of Alexa-labeled CS-EVs for 6 h. **(C)** Representative Z series confocal microscopy images of undifferentiated CaCo-2 cells showing the internalization of CS-EVs. **(D)** Differentiated CaCo-2 cells treated with 5 μg/ml of Alexa-labeled CS-EVs for 6 h **(E)** and 24 h. **(F)** Representative Z series confocal microscopy images of epithelial CaCo-2 cells, showing the internalization of CS-EVs. White scale bars represent 5 μm.

Therefore, to determine the ability of human epithelial cells to internalize CS-EVs, we conjugated CS-EVs with the Alexa Fluor™ 647 dye (Figure 3) to monitor their cellular fate. CaCo-2 cells were grown up to confluence and treated with labeled CS-EVs for 6 h (Figure 4B). Undifferentiated CaCo-2 cells formed multiple layers, and many intra-layer spaces were observed by confocal microscopy (Figures 4A,B). The movie reconstructed from the images (Supplementary File 1) showed that CS-EVs were able to penetrate deeply through the cavities formed by multiple cell layers. As expected, the small dimension of CS-EVs facilitates their diffusion and the further internalization into cells. In fact, to assess this latter aspect, we employed also differentiated CaCo-2 cells (i.e., epithelial cells) that grow mainly as a monolayer. We observed that, after 6 h of incubation with CS-EVs, the differentiated cells presented only a small amount of vesicles within the cytoplasm, whereas, after 24 h, we observed a higher internalization compared to untreated control cells (Figures 4A,B). This time-dependent internalization has been observed also for the undifferentiated CaCo-2 cells (Figures 4D,E), and this process was complete after 24 h. Z-series obtained with confocal microscopy strongly suggested that the CS-EVs were located within their cytoplasm in both undifferentiated (Figure 4C) and differentiated (Figure 4F) cells, supporting the efficient internalization.

### CS-EVs Reduce the Expression of Inflammatory Genes and Enhance That of Permeability-Related Genes

**Figure 5:**
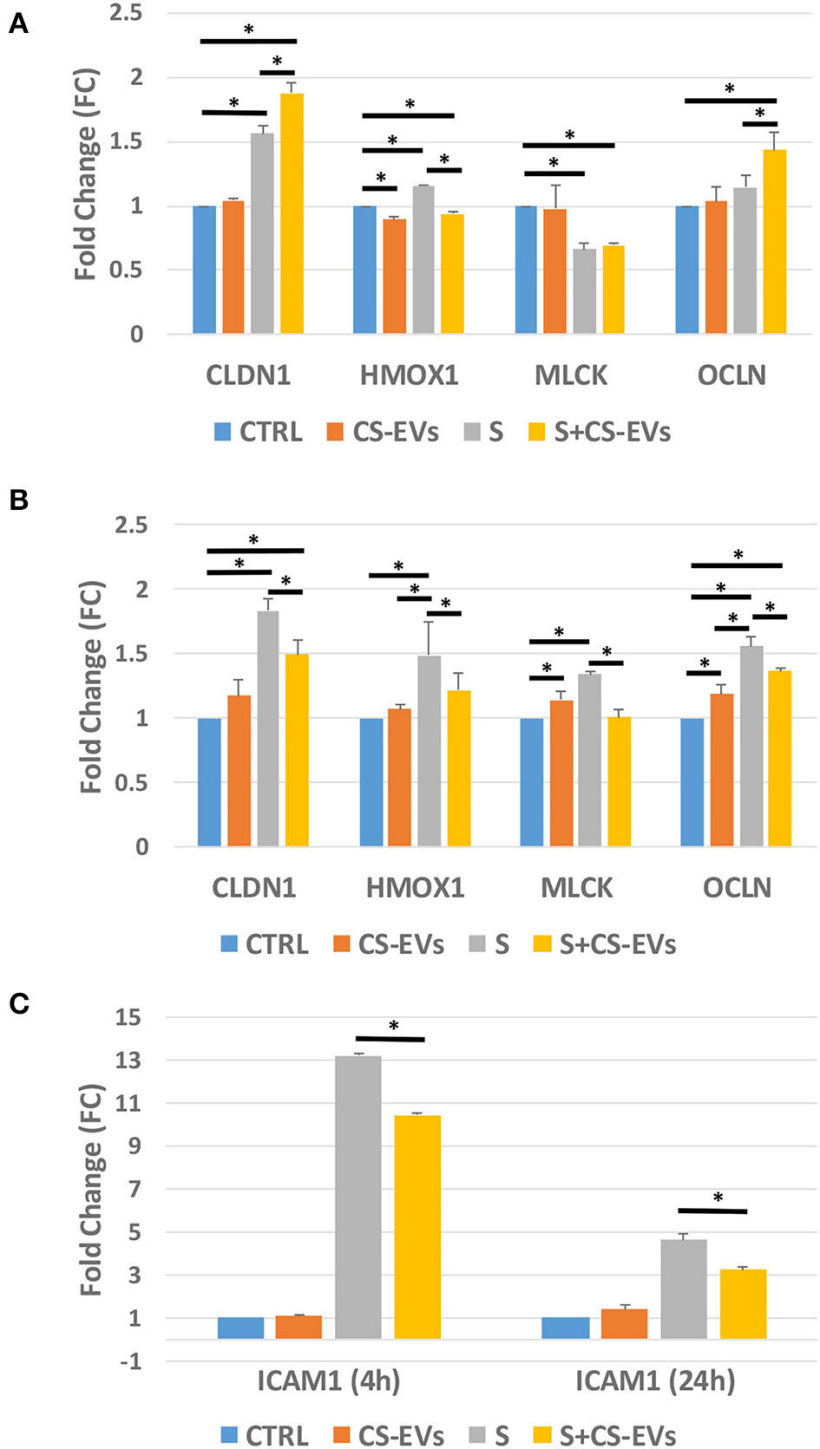
Gene expression of tight junctions and inflammatory genes. Real-time qPCR gene expression analysis of intestinal epithelium genes pretreated (or untreated) with CS-EVs (20 μg/ml) stimulated with an inflammatory cocktail (TNF-α, IL-1β, and IFN-γ, 10 ng/ml each). **(A,B)** show the expression of CLDN1, HMOX1, MLCK, and OCLN genes after stimulation for 6 and 24 h, respectively. **(C)** shows the expression of ICAM-1 after stimulation for 6 and 24 h. Significant differences have been indicated by an asterisk. (* = *p*-value < 0.05).

In the light of using CS-EVs as candidate natural modulators of biological processes related to pro-inflammatory processes or the tightening of intestinal intracellular junctions (i.e., tight junction proteins) that may determine an unbalanced homeostasis and the onset of different gastrointestinal diseases, we investigated the effects of CS-EVs on the gene expression of differentiated CaCo-2 cells as a model of the normal intestinal epithelium (Figure 5). In particular, we focused on the evaluation of tight junction genes (i.e., CLDN-1, CLDN-4, GJB3, OCLN, and ZO-1), inflammatory and immune response genes (i.e., IL-6, ICAM-1, MAPK-1, and TLR8), and other defense mechanism genes (i.e., REG3G, SRC, CTSB, and MLCK) under an inflammatory stimulus that consisted in a cytokines cocktail, with and without a pretreatment with CS-EVs.

Out of the analyzed genes, only CLDN1, OCLN, MLCK, HMOX-1, and ICAM1 resulted significantly dysregulated at 4 h (Figure 5A) and 24 h (Figure 5B) after the treatment. As reported in Figure 5, the pretreatment of CaCo-2 cells with CS-EVs did not generally induce a marked increase of the expression of the considered genes, suggesting that the effect of CS-EVs is negligible. On the contrary, the inflammatory stimulus induced a significant increase of gene expression both at 6 and 24 h compared to controls (Figure 5C). ICAM1 increased its expression more than 12 times at 6 h and remained high up to 24 h. The expression of CLDN1, HMOX-1, and OCLN increased, whereas that of MLCK decreased.

Interestingly, the presence of CS-EVs inhibited the upregulation of ICAM1 or HMOX-1 by the stimulus, suggesting that these vesicles may have a protective role in the inflammatory and oxidative stress pathways activation (Figure 5C). Similarly, the upregulation of CLDN-1 and OCLN that are both involved in tight junction formation has been increased since the beginning (6 h). This suggests that CS-EVs activate rapidly the formation of cell-cell junctions that generally counteract the disruption of the barrier integrity induced by the inflammatory cocktail. Similar conclusions can be drawn for MLCK at 24 h, whereas, at 4 h, both the inflammatory stimulus and the combined treatment with CS-EVs led to a downregulation of this gene.

### CS-EVs Counteract the Occludin Protein (OCLN) Cytoplasmic Distribution After Stimulation With the Cytokine Cocktail

**Figure 6:**
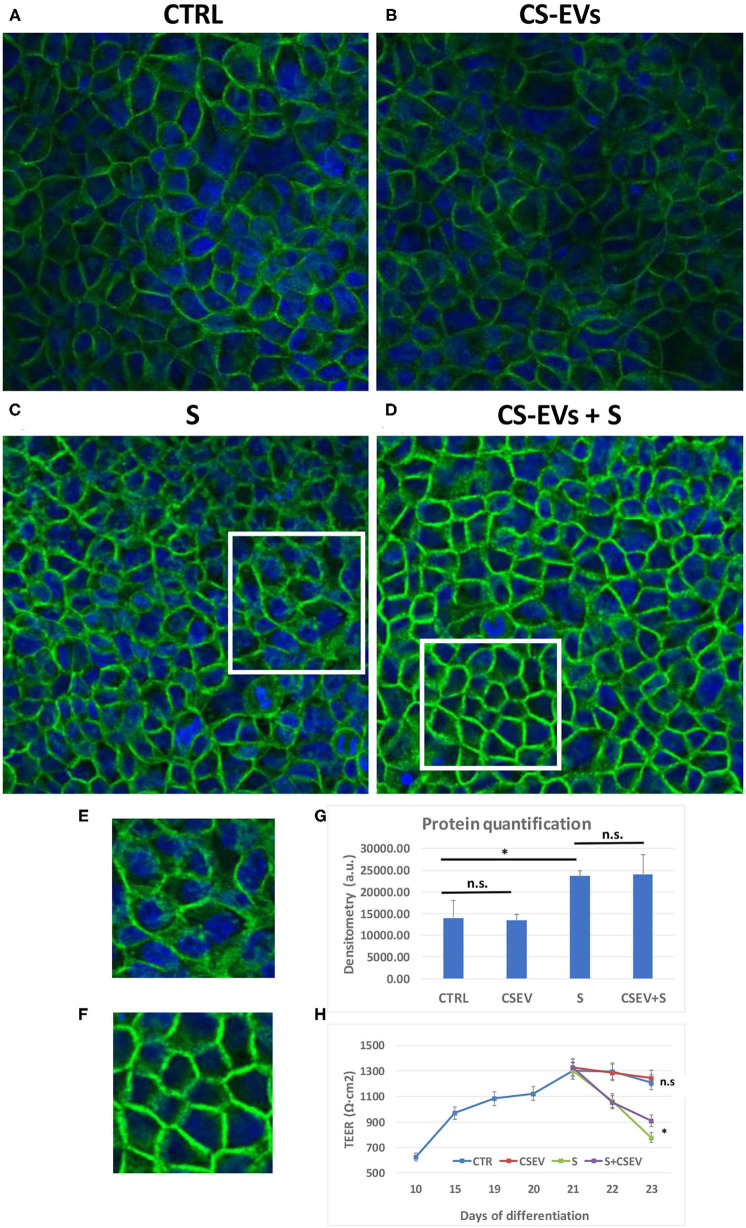
Localization of Occludin within cellular junctions. Differentiated CaCo-2 cells **(A)** were treated with 20 μg/ml CS-EVs **(B)**, a cytokine cocktail (TNF-α, IL-1β, and IFN-γ, 10 ng/ml each) without **(C)** and with a pretreatment with CS-EVs for 1 day **(D)**. The representative magnifications **(E,F)** outlined the different distributions of occludin compared to control cells. In **(G)**, we reported the quantification of fluorescence intensity in the various conditions, whereas, in **(H)**, we reported the TEER measurements in the various conditions (stimulation after complete differentiation). Significant differences have been indicated by an asterisk. (* = *p*-value < 0.05).

To assess the counteracting effect of CS-EVs on the inflammatory stimulus by the cytokine cocktail, we studied the localization of the occludin protein OCLN (Figure 6). To this aim, CaCo-2 cells were cultured and differentiated, pretreated, and stimulated as already described above.

Figures 6A,B show the monolayer of differentiated CaCo-2 cells (with or without the pretreatment with CS-EVs) and after the treatment with the cytokine cocktail (Figures 6C,D). The confocal images confirmed that the pretreatment with CV-EVs did not alter the cell morphology (Figures 6A,B), the localization of OCLN or the amount or the protein (Figure 6G). On the contrary, the stimulation of epithelial cells with the cytokine cocktail (with or without the pretreatment with CS-EVs) impaired its localization (Figures 6C,D) and increased the amount of occludin (Figure 6G) compared with control cells. Moreover, the localization of the occludin protein in epithelial cells pretreated with CS-EVs (stimulated with the cocktail) resulted distributed mainly to the cell membrane (Figure 6D and magnification in Figure 6F), and this difference is statistically significant compared with cells that were not pretreated with CS-EVs (Figure 6C and magnification in Figure 6E). Finally, also, the transepithelial electrical resistance (TEER) measurements (Figure 6H) indicates that the pretreatment with CS-EVs is able to increase significantly the TEER, compared with the untreated epithelial cells. This finding is in agreement with previous observations that indicate that CS-EVs are able to increase the intercellular junctions and support once more the beneficial effects of these fruit vesicles.

## Discussion

In the last few years, a growing interest was focused on the discovery of nanovesicles from edible plants, such as fruits and vegetables (19, 28, 29). The deep characterization of these vesicles revealed the presence of bioactive molecules (i.e., nucleic acids, proteins, lipids, and small RNAs), just like the mammalian exosomes. Since exosomes have been widely recognized to act as messengers between different kinds of cells or organs since their first discovery (30), it appears clear that it is the potential interest in extracellular vesicles of vegetal origin as modulators of important physiological pathways. Moreover, extracellular vesicles derived from different species of plants have different biological effects on the recipient mammalian cells (9, 28, 31), suggesting a potential inter-kingdom communication, although a complete understanding of these mechanisms requires further explorations. Zhang et al. have demonstrated that grape vesicles are taken up by mouse intestinal macrophages, inducing an antioxidant genes expression and the suppression of pro-inflammatory cytokines (9). Despite these few studies, little is known about vesicles from edible foods (i.e., fruits of the Mediterranean diet such as oranges) consumed in our daily diet. Moreover, their functional role in cross-kingdom interaction must be further explored. In our work, we isolated extracellular vesicles from the fresh orange of the *Citrus sinensis* species (variety *Tarocco*) highly consumed in the Mediterranean area. The daily consumption of orange juice is usually associated with an improved diet quality and a beneficial health effect (32, 33).

Our results showed that extracellular vesicles are abundant in the fresh juice, and the biophysical characterization demonstrated that CS-EVs are dimensionally homogeneous (~70 nm in diameter). Furthermore, we simulated the environment of the gastrointestinal tract *in vitro* by the use of proper solutions to see if CS-EVs are stable and viable after the pH change of the gastrointestinal tract. We showed that there are no significant variations in the size of CS-EVs after 2 h from the initial incubation, outlining the great stability of the vesicles in these conditions. We noticed only a slight increase in vesicles dimension during the passage through the different solutions, maybe attributable to an increase of the pH values that may lead to a different charge polarization (i.e., neutralization) of the EVs membranes. However, our *in vitro* data demonstrated that CS-EVs can reach the lower gastrointestinal tract with their intact cargo. This result confirms that CS-EVs can be considered stable carriers through all of the gastrointestinal tract and potentially employed as useful and innovative biocarriers.

The hypothesis we wanted to demonstrate was that the ingestion of CS-EVs can be absorbed/internalized by the intestinal epithelial cells and exploit a “beneficial” effect (i.e., antioxidant effects or the restoring of increased intestinal permeability). In humans, the damaged intestinal epithelium (i.e., leaky gut) is the source of many metabolic and chronic diseases (34, 35). Therefore, the prevention of these damages or their restoration could be beneficial and ultimately lead to a healthier lifestyle.

To investigate these effects on a human intestinal epithelium, we employed CaCo-2 cells that are able to spontaneously form a differentiated cell monolayer after culturing them for 21 days. We employed also a model of overgrown undifferentiated cells that are able to form multilayers in order to investigate the ability of CS-EVs to pass through intercellular spaces. To visualize CS-EVs, we functionalized the amino groups located on their membrane surface with an efficient Alexa fluorescent dye by exploiting an N-hydroxysuccinimidyl ester (NHS) conjugation (Figure 3).

Compared with other traditional dyes used for EVs labeling, Alexa Fluor 647 presents several advantages: (1) the intrinsic brightness of this marker allows optimal setting parameters for confocal microscopy and can be easily detected due to its high extinction coefficient; (2) this dye, owing to its simple chemical structure, cannot form micelles or other aggregates that can fluoresce under the microscope and cause false positive results in EVs uptake experiments, owing to an increase in the EVs size, and this is an advantage over PKH26 dye (36). This bioconjugation reaction allowed us to obtain an original compound, possessing a stable labeling moiety that we reported here for the first time. This easy and cheap protocol can be also used for many other purposes in the field of EVs.

As expected, we confirmed that CS-EVs were not toxic to intestinal epithelial cells and that they are able to enter into cells just after 6 h of incubation. We found that the internalization of CS-EVs into intestinal epithelial cells is able to modulate the expression of important genes related to inflammatory pathways, such as HMOX-1 and ICAM1, or to the restoration of intestinal permeability related such as claudins and occludin. Although the number of analyzed genes/pathways was quite limited to unravel all the possible interactions and mechanisms induced by CS-EVs, from the results we obtained, we can strongly suggest that CS-EVs may exert a beneficial effect on human cells and, in particular, intestinal cells. The effects we observed are related to the restoration of tight junctions or to the inhibition of inflammatory genes, but we can hypothesize that these effects are not limited only to those genes and biological pathways. A recent review paper summarized and provided robust evidence, favoring the anti-IBD effects of citrus fruits and their flavonoids in the prevention and treatment of IBD (37).

Although we know that the results obtained in this work are preliminary to more detailed investigations, we foresee that the comprehension of the potentialities of CS-EVs should include also the characterization of their protein and RNA content, a goal that we will continue investigating.

Finally, although partial and focused only on vegetal extracellular vesicles, our data confirm the larger concept that the effects of eating food (with all of its components) belonging to the Mediterranean diet are beneficial to human health.

## Data Availability Statement

The raw data supporting the conclusions of this article will be made available by the authors, without undue reservation.

## Author Contributions

SB and AP designed the study and perfomed the experiments. VD'O and AS performed microscopic analyses. SB, SS, and FB drafted the manuscript. AM drafted and edited the manuscript, checked the data analysis, and supervised the work. All authors contributed to the article and approved the submitted version.

## Conflict of Interest

The authors declare that the research was conducted in the absence of any commercial or financial relationships that could be construed as a potential conflict of interest.

## Publisher's Note

All claims expressed in this article are solely those of the authors and do not necessarily represent those of their affiliated organizations, or those of the publisher, the editors and the reviewers. Any product that may be evaluated in this article, or claim that may be made by its manufacturer, is not guaranteed or endorsed by the publisher.
