# Supplementary material for: Extracellular Vesicles Derived From Citrus sinensis Modulate Inflammatory Genes and Tight Junctions in a Human Model of Intestinal Epithelium
Source: Front Nutr. 2021 Nov 24;8:778998. doi: 10.3389/fnut.2021.778998 (PMC8652296; doi:10.3389/fnut.2021.778998)
Supplement: Supplementary file 1 [file Table_1.docx]

**Supplementary Table (S1)**

Primer sequences used for RT-qPCR.

| **Gene** | **Primer sequences** | **Assay ID** |
| --- | --- | --- |
| GUSB | 5’-TCACTGAAGAGTACCAGAAAAGTC-3’  3’-GCTCTCACGACCCCTTATTTT-5’ | Hs.PT.39a.22214857 |
| IL-6 | 5’-GCTGCTTTCACACATGTTACTC-3’  3’-TAAGCAAGACTTCTCCACTCAC-5’ | Hs.PT.49a.20968536 |
| ICAM-1 | 5’-GCTATTCAAACTGCCCTGATG-3’  3’-CGTTCTTGGAATGGGATGCG-5’ | Hs.PT.58.4746364 |
| MAPK-1 | 5’-CATTCAGCTAACGTTCTGCAC-3’  3’-CGTCTAGGTCTGGTACTAGTG-5’ | Hs.PT.58.39782850 |
| CLDN-1 | 5’-CAGCATGGTATGGCAATAGAATC-3’  3’-GACGAAGAGAGACGGAAGAC-5’ | Hs.PT.58.39440567 |
| CLDN-4 | 5’-CCATATAACTGCTCAACCTGTCC-3’  3’-CGTAGTCCTGACCGAAATAGA-5’ | Hs.PT.58.1326185.g |
| OCLN | 5’-AGGCGAAGTTAATGGAAGCTC-3’  3’-CGAACAGTAAGTGAAACGGTA-5’ | Hs.PT.58.15235048 |
| TJP1 | 5’-GCTGGCTTATTCTGAGATGGA-3’  3’-CTTACATACACCTCTCTGCGC-5’ | Hs.PT.58.2456962 |
| GJB3 | 5’-ATAATGTGTAAGAGAGGTGAGAAGT-3’  3’-TCTGGATTCCGTAGAGATGAGG-5’ | Hs.PT.58.39590773.g |
| REG3G | 5’-GCATACCAGATCTCACCAGAG-3’  3’-CAGTCCAAGTTCCACTTCTTTG-5’ | Hs.PT.58.50445678.g |
| SRC | 5’-CAATGCAGAGAACCCGAGAG-3’  3’-GTTGCACTTCGTGATGTTCTAG-5’ | Hs.PT.58.19132577 |
| CTSB | 5’-GATCTGCATCCACACCAATG-3’  3’-CGAACCTTGAAGACCTGTTCT-5’ | Hs.PT.58.1907042 |
| MYLK | 5’-TCCTGAAGTGATCAACTATGAGC-3’  3’-AGTACCCTCTGTTGCTATTGC-5’ | Hs.PT.58.194304 |
| TLR-8 | 5’-GATCCAGCACCTTCAGATGAG-3’  3’-CCATAGCTTCAACCCAGTTCA-5’ | Hs.PT.58.15023918.g |
